# Three dimensional digital reconstruction of the jaw adductor musculature of the extinct marsupial giant *Diprotodon optatum*

**DOI:** 10.7717/peerj.514

**Published:** 2014-08-14

**Authors:** Alana C. Sharp

**Affiliations:** School of Earth, Atmosphere and Environment, Monash University, Clayton, Victoria, Australia

**Keywords:** Marsupial, Diprotodontia, Functional anatomy, Digital reconstruction

## Abstract

The morphology and arrangement of the jaw adductor muscles in vertebrates reflects masticatory style and feeding processes, diet and ecology. However, gross muscle anatomy is rarely preserved in fossils and is, therefore, heavily dependent on reconstructions. An undeformed skull of the extinct marsupial, *Diprotodon optatum*, recovered from Pleistocene sediments at Bacchus Marsh in Victoria, represents the most complete and best preserved specimen of the species offering a unique opportunity to investigate functional anatomy. Computed tomography (CT) scans and digital reconstructions make it possible to visualise internal cranial anatomy and predict location and morphology of soft tissues, including muscles. This study resulted in a 3D digital reconstruction of the jaw adductor musculature of *Diprotodon*, revealing that the arrangement of muscles is similar to that of kangaroos and that the muscle actions were predominantly vertical. 3D digital muscle reconstructions provide considerable advantages over 2D reconstructions for the visualisation of the spatial arrangement of the individual muscles and the measurement of muscle properties (length, force vectors and volume). Such digital models can further be used to estimate muscle loads and attachment sites for biomechanical analyses.

## Introduction

Understanding the relationship between form and function is the aim of biomechanical analysis of extinct or extant vertebrates (Lauder, 1995). Soft tissues can be preserved in the fossilised remains, but in the majority of cases it is necessary to infer the arrangement of muscles and ligaments from skeletal remains in order to predict muscle function, skeletal movement, and possible behaviour (Lauder, 1995; Witmer, 1995). Using the Extant Phylogenetic Bracket (Witmer, 1995), living taxa can offer valuable insights into the arrangement of muscles for closely related extinct taxa. However, where there are no close living analogues, palaeontologists must rely on fossil material alone to reconstruct soft tissues. This in turn requires a broad knowledge of vertebrate anatomy.

In the past, bone and soft tissue structures were described and illustrated using line drawings in standard anatomical planes and often only one or two diagrams were given in the published description. It is difficult to get a full understanding of the spatial arrangement of complex three-dimensional (3D) structures when illustrated in two dimensions, leading to misunderstandings, or misinterpretations of the literature. In recent years, imaging techniques, such as computed tomography (CT) and magnetic resonance imaging (MRI), have facilitated 3D reconstruction and visualisation of jaw muscles and are becoming more common in the analysis of extinct (Lautenschlager, 2013; Snively et al., 2013; Snively & Russell, 2007) and extant taxa (Cox & Faulkes, 2014; Cox & Jeffery, 2011; Holliday et al., 2013; Lautenschlager, Bright & Rayfield, 2014; Quayle et al., 2014).

For this study, the jaw adductor musculature of the extinct *Diprotodon optatum*, the largest marsupial known, has been reconstructed. Like other members of the Australian megafauna, *Diprotodon* became extinct during the Late Pleistocene. The family Diprotodontidae, in which *Diprotodon* belongs, is a diverse group of vombatomorphian (wombat-like) marsupials with no close living representatives. The living relatives closest to *Diprotodon* include koalas (Family Phascolarctidae) and wombats (Vombatidae) (Murray, 1991). However, none of these living species possesses a cranium that is a good analogue to the unique cranial structure of *Diprotodon*. Thus, direct comparisons cannot be made between the diet and ecology of these living taxa to that of *Diprotodon*. The jaw muscles of interest in this study include the temporalis, masseter and pterygoid muscles, all of which play a role in closing the jaw. This reconstruction is the first 3D digital reconstruction for a marsupial megafauna species.

The reconstruction provided in this study is used to predict the jaw movements and diet of *Diprotodon*. Specifically, it is hypothesised that the arrangement of masticatory muscles and skull morphology will impact biomechanical performance during feeding. This arrangement may reflect an adaptation to a particular diet, specifically that of grazers or browsers. Modern marsupials, including koalas, wombats and macropods, are used to draw comparisons with osteological correlates visible on the fossilized bone. Based on this initial reconstruction, a broader biomechanical analysis of skull function between these species has been carried out.

## Materials and Methods

### Nomenclature

The terminology used here was selected for its consistency with jaw muscle nomenclature used for marsupials (Abbie, 1939; Crompton et al., 2008; Davison & Young, 1990; Murray, 1998; Tomo et al., 2007; Turnbull, 1970; Warburton, 2009). The masseter muscle group includes three portions (superficial, deep and zygomaticomandibularis) based on their area of origin. Each portion can include multiple layers within it if partial laminae are present. However, in fossil taxa one cannot ascertain the complexity of the muscles, so each portion of the masseter has been modelled as a single muscle. The temporalis has been modelled as consisting of two layers (superficial and deep), based on muscle scars preserved on the bone. The pterygoid muscles are referred to as medial and lateral following terminology of Abbie (1939).

### Data acquisition and processing

An undeformed cranium of *Diprotodon optatum* (NMV P31299) recovered from Pleistocene sediments at Bacchus Marsh in Victoria, Australia (37°40′S, 144°26′E) was scanned using computed tomography (CT) with a Siemens Sensation 64 scanner (Siemens Medical Solutions) at St. Vincent’s Public Hospital, Melbourne. The specimen was scanned with 0.6 mm slice thickness and a 0.3 mm interslice distance to produce 978 slices. Although both zygomatic arches are fractured the specimen represents the most complete and best preserved cranium of the species known, offering the best opportunity for a complete reconstruction of this taxon.

Two near-complete lower mandibles (NMV P151802 and NMV P157382), recovered from Bacchus Marsh, were also CT scanned and reconstructed to be used as proxies for a complete skull reconstruction. These two lower mandibles were approximately the same size so only minimal scaling was needed to reconstruct the complete lower jaw. Tooth eruption and wear patterns indicate that the *Diprotodon* fossils recovered from the Bacchus Marsh assemblage is dominated by sub-adult to young adult individuals (Price, 2008). Therefore, the mandible also required minimal scaling to match the size of the cranium.

### Building the model

**Figure 1:**
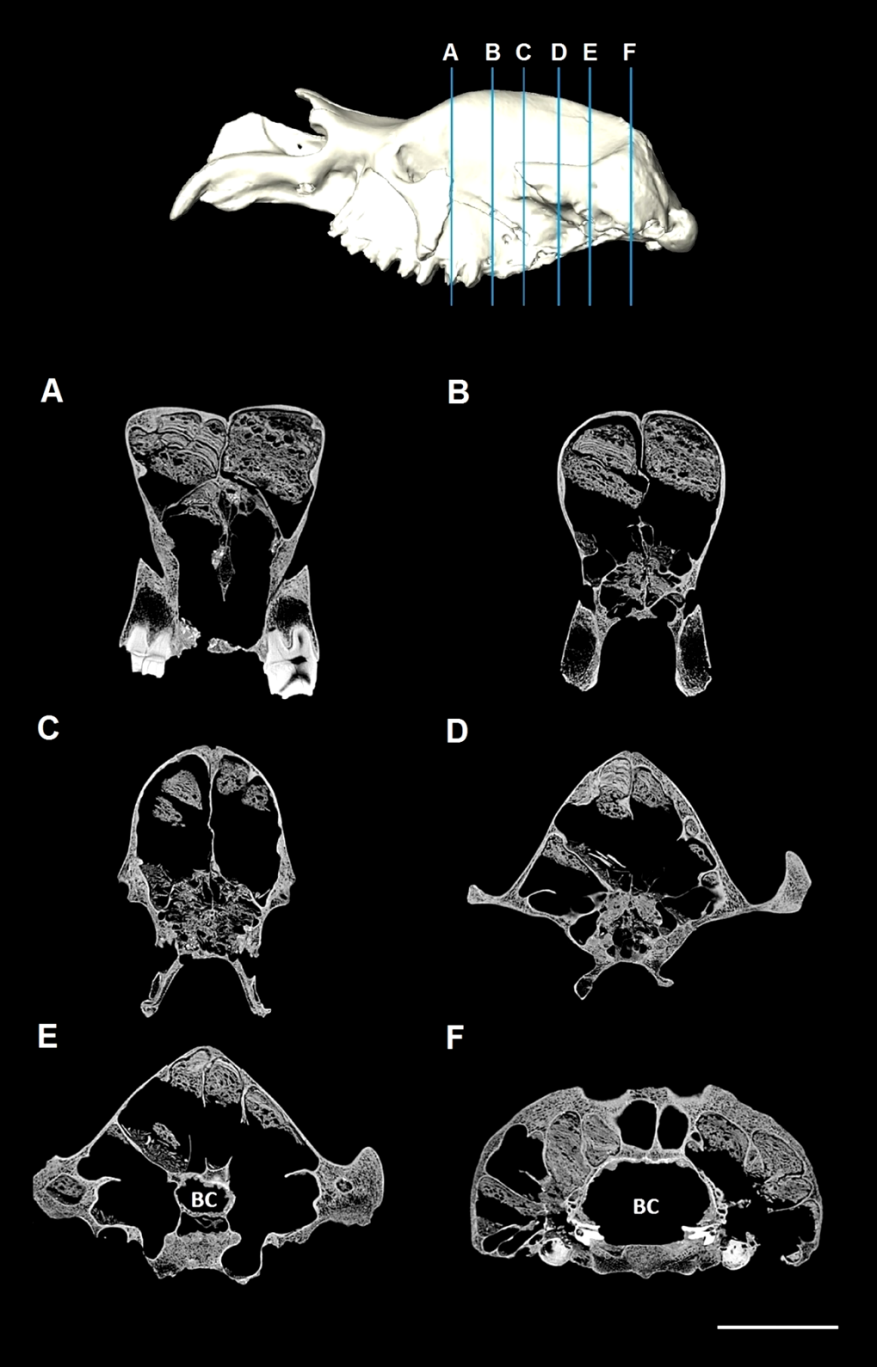
CT slices of *Diprotodon*. Coronal CT slices of *Diprotodon* cranium (NMV P31299) showing the extent of the sinus that extends from the frontal bone to the occipital bone (A–F) and their respective locations shown on the 3D model above. Note the presence of the rock matrix within the sinuses. BC, Brain case. Scale bar is 10 cm.

The CT data were imported into the image processing software program Mimics 13.1 (Materialise), where editing of the CT slices took place. Many of the internal cavities, including the extensive sinuses within the fossilised cranium, were filled with inorganic matrix (Fig. 1). This matrix has approximately the same radiodensity (transparency to the passage of X-rays) as the fossilised bone; therefore, automated thresholding (the automated segmentation process of selection and isolation of the structure of interest based on its grey values) could not be used to remove the matrix. Manual segmentation was performed in all three planes to remove this inorganic matrix, clearly identifiable by small gaps between fossil bone and rock matrix (Fig. 1). Delicate internal struts that had noticeably been broken were also reconstructed manually. Finally, a 3D surface reconstruction was exported in stereolithography (STL) format.

The surface model was then imported into the program Geomagic Studio 10.0 (Geomagic, Inc.), which allowed editing to further improve the quality of the reconstruction. The preliminary model required reconstruction of fragmented areas, smoothing and removing unnecessary non-structural anatomy, such as the delicate nasal turbinates that were partially preserved, but unnecessary for this study. Breaks and missing regions of the cranium, including the zygomatic arches, were reconstructed with the mirroring tool, superimposition and curve-fitting functions of more complete areas, as well as reference from previous reconstructions and other skull specimens (NMV P150021). In all steps involving modification, careful consideration was taken when editing the geometry to maintain biological accuracy.

The CT data for each mandible were imported separately into the image visualisation and processing software program Avizo (Versions 6.3 and 7.0, Visage Imaging, Inc.), where a combination of automated thresholding and manual editing were used to produce two 3D surface reconstructions. Both surface reconstructions were exported as STL (*.stl) files and imported into Geomagic Studio, where a combinations of tools, including reflection, superimposition and scaling, were used to produce a reconstruction of a complete mandible.

The reconstructed mandible and cranium were then aligned in Geomagic, exported as one surface model and imported into Avizo to digitally reconstruct the jaw adductor musculature.

### Soft tissue

The complete reconstructed and aligned model of the *Diprotodon* skull was first converted from an STL file to a series of slices using the “ScanConvertSurface” module in Avizo. The muscular anatomy was constructed following a similar method to Lautenschlager (2013) and Curtis et al. (2009).

The jaw adductor musculature of *Diprotodon* was reconstructed using data from dissections of modern wombats, koalas and kangaroos, MRI scans of a wombat and koala, and previously published dissections (Crompton et al., 2008; Davison & Young, 1990; Murray, 1998; Tomo et al., 2007; Warburton, 2009). This allowed the reconstruction of the complex muscle anatomy by assessing the locations of muscle attachment sites, muscle volume and identifying muscle orientations. Muscle origin and insertion sites for *Diprotodon* could be identified for each muscle based on surface features such as muscle scars, depressions, ridges, crests, processes or other bone morphology.

To visualise the gross muscle topology and orientation, models were first constructed using simplified cylinders to connect the origin and insertion sites for each muscle, similar to the methodology of Curtis et al. (2009). Based on these simplified cylinders and the constraints imposed by the bone, muscles were then fully “fleshed-out”. Each muscle was constructed first in the coronal plane and then edited in the remaining two planes, sagittal and horizontal, for biological accuracy. The volume of each muscle was estimated based on the area of the muscle attachment sites and the constraints provided by the bone. For example, the available space imposed by the structure of the bones, in particular the area of the temporal fossa, coronoid process and inflected angle of the mandible provided constraints on the possible volume of each muscle. If the origin or insertion of one muscle was not clear, the extent of that muscle attachment site was based on the location of one or two adjacent muscles with clearly identified muscle attachment sites. The extent of the superficial masseter was based on the bulky superficial masseter in wombats (Crompton et al., 2008; Murray, 1998) due to the large area of insertion on the ventral surface of the inflected angle of the mandible, not present in koalas. It was reconstructed with the same shape, and approximately the same thickness as the superficial masseter in wombats based on the MRI data. However, the full extent of this muscle cannot be entirely estimated and the reconstruction in this study may be conservative. The volume, in millimetres squared, for each reconstructed muscle was obtained using the Avizo “MaterialStatistics” module, and the angles of fibre direction were measured directly from the 3D model using the Avizo measurement tool.

## Results

### Craniodental anatomy

A comprehensive description of the bony anatomy of the masticatory system, including dentition, dentary and cranium, is provided by Price (2008) and Owen (1870). A general description will be given here to highlight elements that are the most important for this study.

### Dentition

The Diprotodontidae, which includes a diverse group of vombatomorphian marsupials, are characterised by relatively simple bilophodont molars, oval to triangular P^3^ in dorsal aspect, and six upper and two lower incisors. Members of the Subfamily Diprotodontinae, to which *Diprotodon* belongs, are unique in having a characteristic horseshoe-shaped lophodont pattern of the P^3^ when the crown is worn (Murray et al., 2000). In *D. optatum* the single pair of lower incisors are chisel-like, straight to slightly curved dorsally, and occlude with the posterior surface of I^1^ and the horizontal surface of I^2^ and I^3^. The lower incisors are rootless and continue to grow throughout the animal’s life, as is the case in modern wombats and kangaroos. The upper first incisors are also rootless, like wombats, and are long and curved ventrally. The molar teeth are bilophodont, similar to those in *Macropus*, and increase in size from M1 to M4.

### Dentary

The mandible is elongate, ranging from approximately 500 to 650 mm (Price, 2008). The symphysis is fused and elongate, accounting for roughly one third of the length of the dentary and accommodates the long open-rooted lower incisors. The coronoid process is tall and slender. The posterior edge of ascending ramus is constricted at the neck of the condyle, which is wide medio-laterally and isolated high above the occlusal tooth line. The articular surface is convex posteriorly and anteriorly inclined. The angular process also projects far dorsal to the occlusal tooth surface.

### Cranium

**Figure 2:**
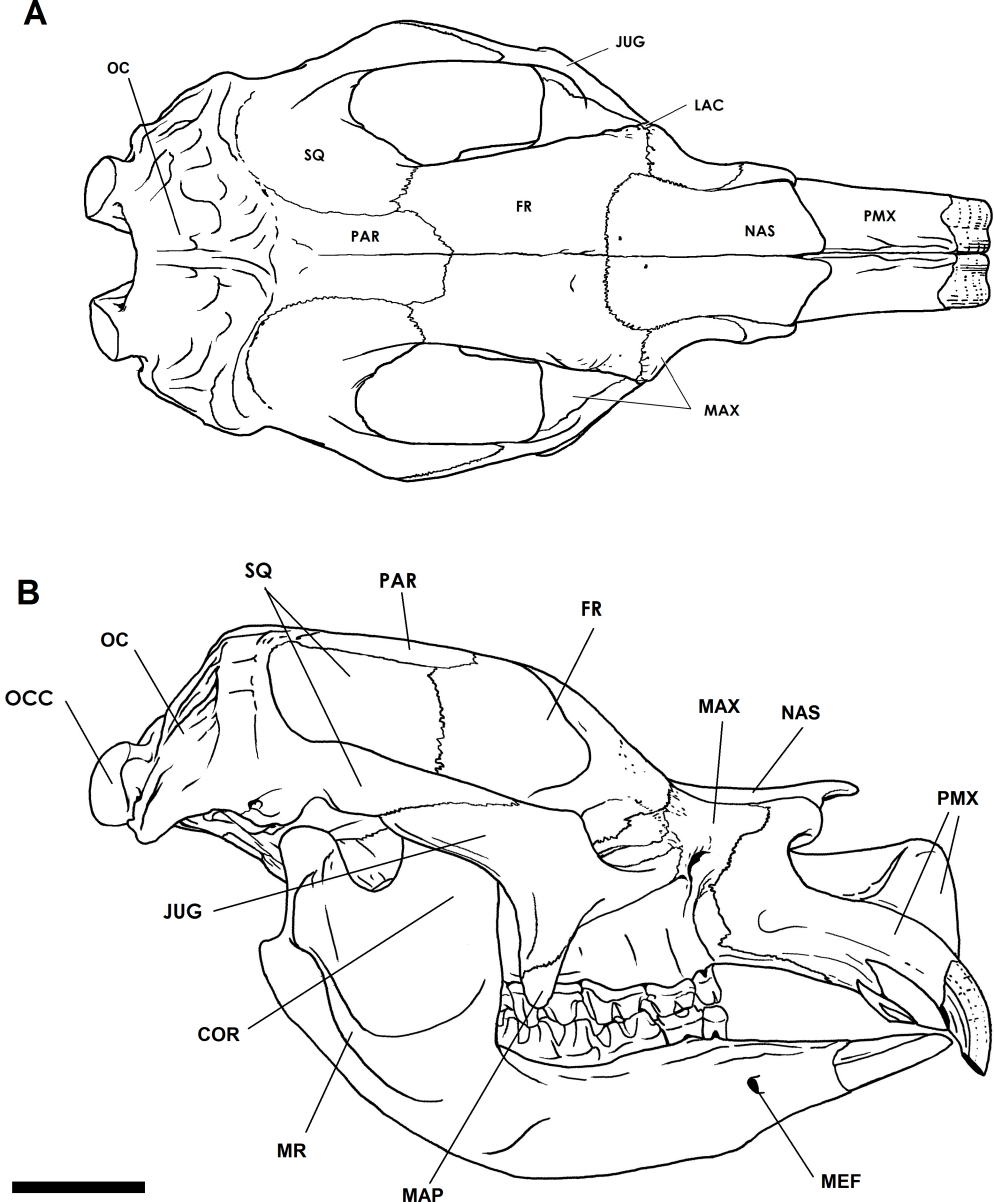
Cranial anatomy of the Bacchus Marsh *Diprotodon*. Dorsal view (A) and lateral view including the mandible (B). Scale bar = 10 cm. Abbreviations: COR, coronoid process; FR, frontal; JUG, jugal; LAC, lacrimal; MAP, masseteric process; MAX, maxillary; MEF, mental foramen; OC, occipital; OCC, occipital condyle; PAR, parietal; MR, masseteric ridge; PMX, premaxillary; SQ, squamosal. Illustration by P Trusler courtesy T Rich, Museum Victoria and the ARC.

Adult crania range from 650 mm to 1,000 mm in condylobasal length (Owen, 1870; Price, 2008). In lateral aspect, the profile of the cranium is low and elongate (Fig. 2A). In dorsal aspect, the profile is narrow and elongate (Fig. 2B). The frontal is expanded into a pair of crests with large sinuses below. The external nasal cavity is expanded, and bisected by an upward extension of the medial nasal plate of each premaxillary. In addition, the nasal cavity is divided by a complete bony septum. The cranial base is flat and elevated above the level of the occlusal tooth surface. The zygomatic arches are elongate and flare outwards as smooth curves in dorsal aspect, and appear to be flat-sided and angular in ventral aspect. The masseteric process is well developed and deep. The occipital region, instead of being vertically oriented, as in kangaroos, or angled posteriorly as in wombats and koalas, slopes anteriorly from the occipital condyles at an angle of about 60° to the basicranial axis. The occipital condyles are large, 60 mm to >110 mm in depth in adults, and are separated by a gap of approximately 25 mm and 50 mm at the ventral and dorsal surfaces respectively (Owen, 1870).

### Endocranial sinuses

**Figure 3:**
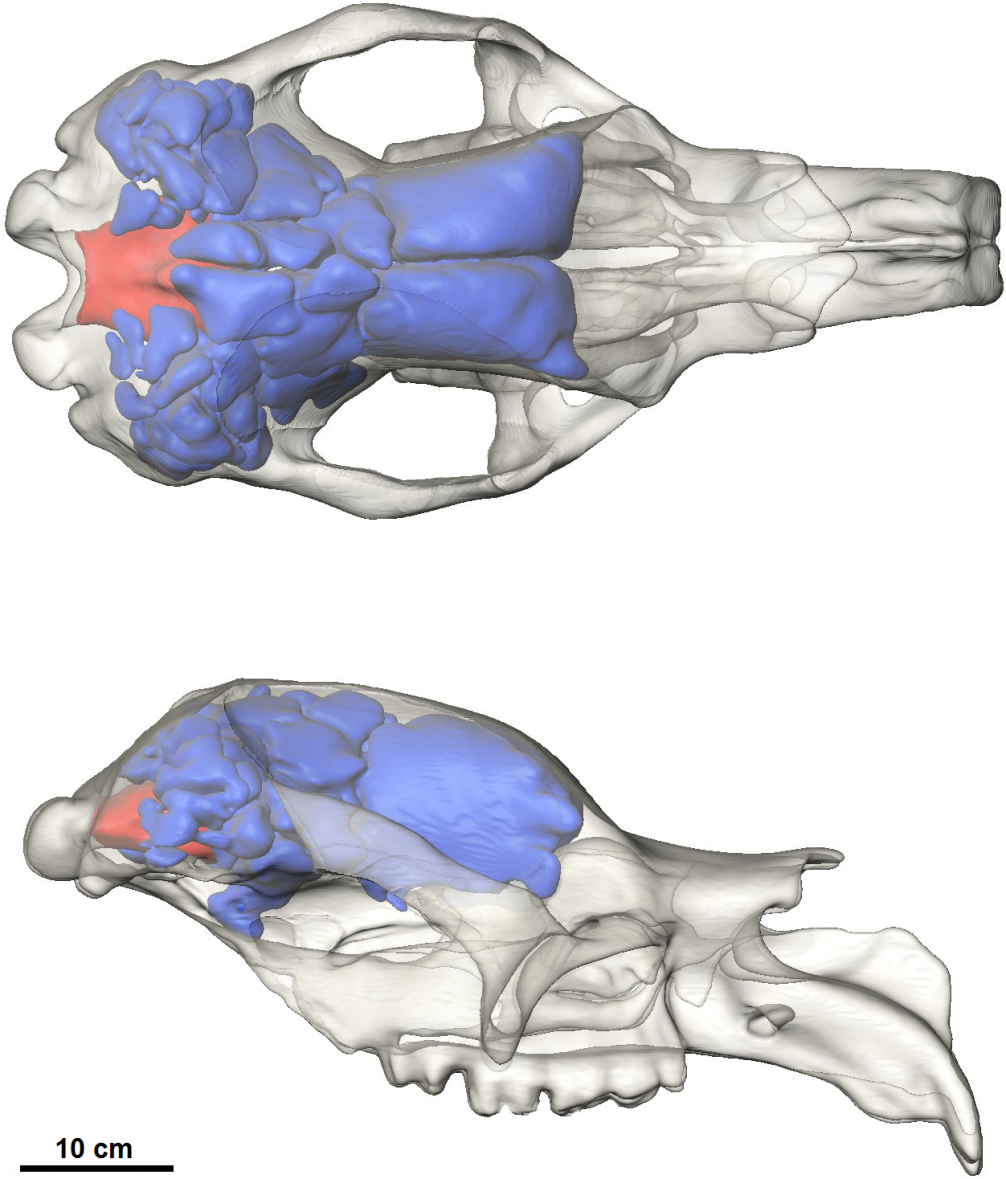
Dorsal and lateral view of the cranial reconstruction of the Bacchus Marsh *Diprotodon*. The sinuses are displayed in blue and cranial endocast in red. Bone is 70% transparent.

The most prominent feature of the endocranium in *Diprotodon* is the extensive endocranial sinuses which extend throughout a large portion of the skull (Fig. 3). The sinuses in the Bacchus Marsh *Diprotodon* are grossly enlarged with a volume of 2,797 cm^3^.

The cranial sinuses of *Diprotodon* are found in the frontal bone lying just anterior to the dorsal chonchal sinus, and extend caudally into the parietals and interparietals, laterally into the squamosals, dorsally over the endocranial cavity and into the occipitals. Anteriorly, the braincase is separated from the external surface of the skull by a series of large frontal sinuses. Laterally and dorsally the braincase is surrounded by epitympanic sinuses, squamosal sinuses and parietal sinuses. The sinuses house numerous plate-like trabeculae (Fig. 1) that span the inner and outer bony mantles as the diploe, an intervening spongy bone layer, which expands during sinus development. A mid-sagittal bony septum is present, extending from the nasals to the occipitals.

The frontal sinus of *Diprotodon* is particularly spacious and relatively simple, divided by only two bony septa, one on the sagittal plane (dividing the sinus into left and right parts) and another in the coronal plane (dividing the sinus into anterior and posterior parts). These partitions correspond to the sutures between the frontals and parietals (coronal suture), and the midsagittal suture. The posterior parietal and squamosal sinuses are further subdivided forming a complex, interconnected network of chambers surrounding the middle ear cavity and braincase.

### Musculature

#### Masseter complex

**Figure 4:**
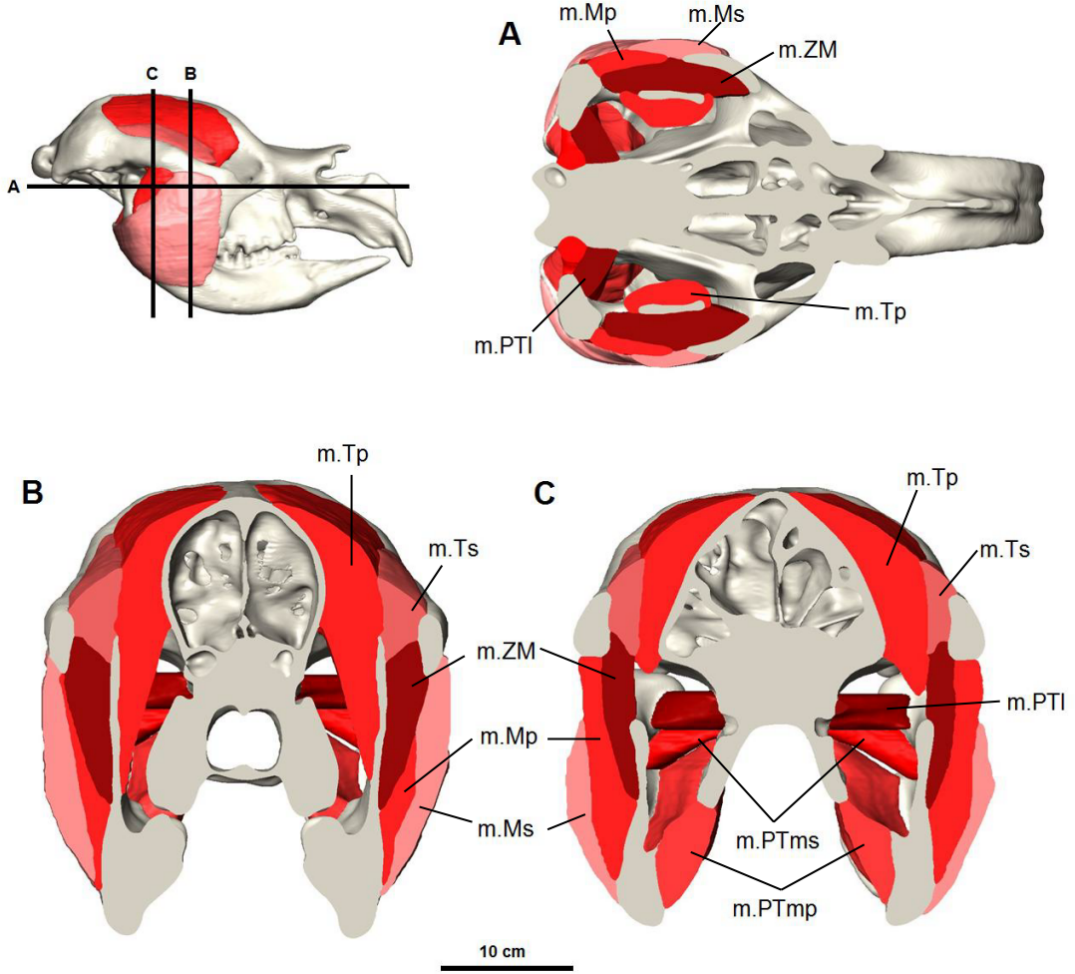
Digital reconstruction of the jaw adductor musculature in the Bacchus Marsh *Diprotodon*. (A) horizontal and (B, C) coronal cross-sections. Abbreviations: m.Mp, deep masseter; m.Ms, superficial masseter; m.ZM, zygomaticomandibularis; m.Tp, deep temporalis; m.PTl, lateral pterygoid; m.PTms, superficial medial pterygoid; m.PTmp, deep medial pterygoid.

**Figure 5:**
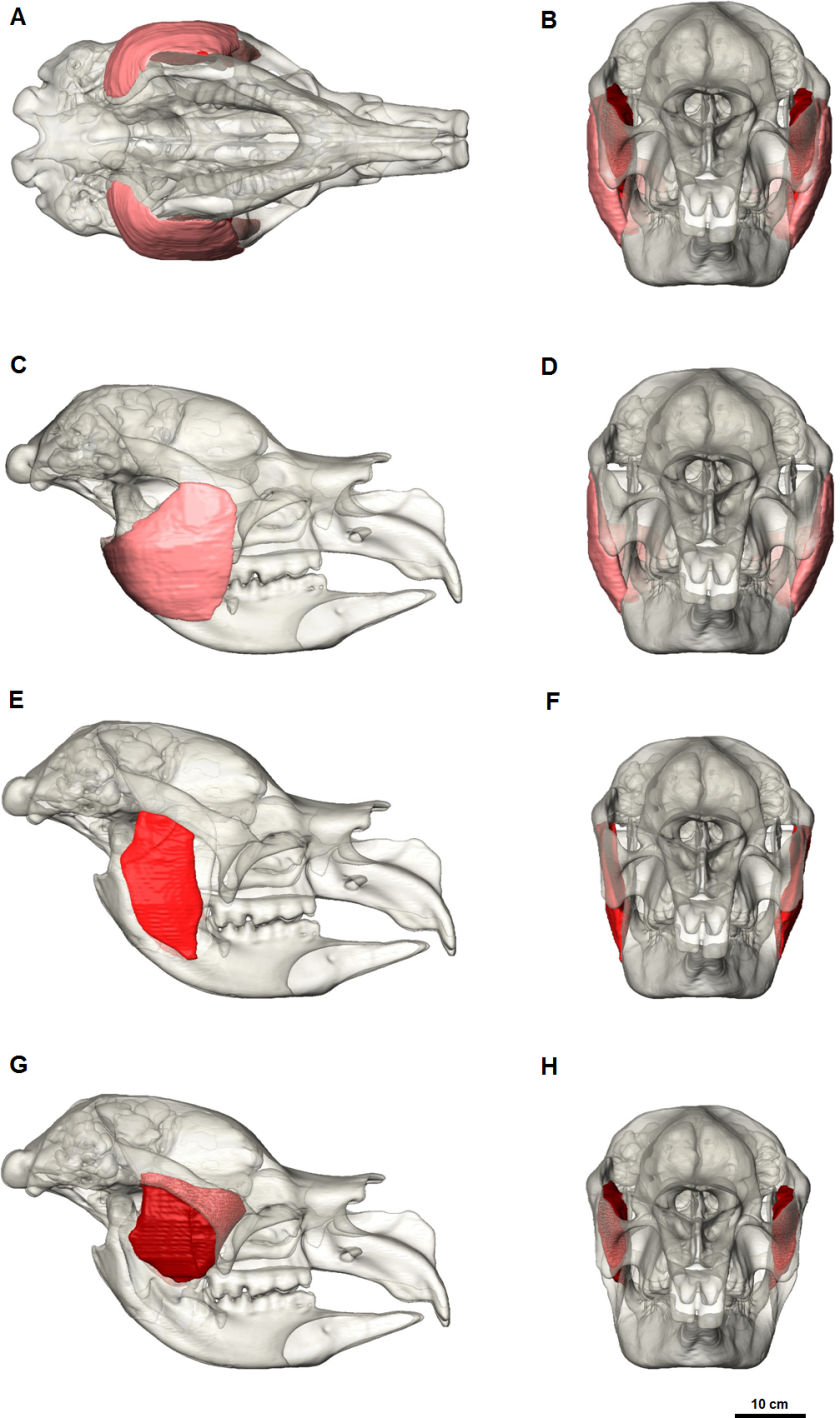
Masseter muscle reconstruction for *Diprotodon*. Ventral view (A) and anterior view (B) of the three masseter muscle layers. (C, E, G) Lateral views; (D, F, H) Anterior views; (C, D) Superficial masseter; (E, F) Deep masseter; (G, H) Zygomaticomandibularis. Bone is 70% transparent.

The masseter muscle was reconstructed in three parts (Figs. 4 and 5) based on dissections of living marsupials conducted for this study and published dissections of wombats (Crompton et al., 2008; Murray, 1998), koalas (Davison & Young, 1990) and kangaroos (Abbie, 1939; Tomo et al., 2007; Warburton, 2009). The masseter muscle group is the largest jaw muscle calculated for *Diprotodon*, accounting for 44 percent of the total jaw muscle mass (Table 1). The principal action of the masseter is to elevate the mandible, with a lesser role in the lateral and protrusive movements during mastication.

*M. masseter superficialis* (m.Ms) has been reconstructed as a single muscle in *Diprotodon* (Figs. 5C and 5D). There is no clear indication based on the fossil bones of multiple tendinous attachments. The superficial masseter originates from a small area on the lateral extremity and the ventral tip of the masseteric process, located above the M^3^ and posterior to the orbit (Fig. 2). The origin is defined by a weak muscle scar, extending from the postero-lateral surface of the masseteric process and along the lateral surface of the anterior two-thirds of the zygomatic arch, anterior and ventral to the jugal-squamosal suture. The muscle fibres would have run postero-ventrally at an angle of approximately 35° to the horizontal. The insertion is large, covering the entire postero-ventral surface of the broad inflected angle of the mandible and the lateral surface of the horizontal ramus.

In kangaroos and wombats the superficial masseter is usually depicted as having two or more parts (Abbie, 1939; Crompton et al., 2008; Tomo et al., 2007). In kangaroos the more superficial layer originates from the small ventral tip of the masseteric process and inserts on the postero-ventral surface of the inflected mandibular angle. The deeper layer originates from the lateral edge of the zygomatic arch and inserts on the lateral surface of the horizontal ramus. These two layers have different fibre directions and provide force in different vectors, allowing better control of motion at the incisors. Without clear muscle scars on the fossilised bone, one cannot determine whether *Diprotodon* had a similar arrangement. However, it is clear that the mandibular symphysis was firmly fused in *Diprotodon* so fine control at the incisors may not have been possible in *Diprotodon*, unlike kangaroos where independent movement of each hemimandible is possible. In the reconstruction presented here, the anterior-lateral origin and ventral-lateral insertion of the superficial masseter would still provide elevation of the mandible allowing the crushing function of molars and protraction of the mandible for occlusion of the incisors to gnaw, crop and cut vegetation.

*M. masseter profundus* (m.Mp) originates along the ventral surface of the zygomatic arch and runs almost vertically to insert on the lower half of the lateral surface of the ascending ramus and into a shallow basin between the masseteric ridge and the ascending ramus (Figs. 5E and 5F). The area of origin on the ventral surface of the posterior half of the zygomatic arch is broad, but only extends along two-thirds of the zygomatic arch narrowing anteriorly on the jugal. The deep masseter elevates the mandible.

*M. zygomaticomandibularis* (m.ZM) originates from the medial surface of the zygomatic arch on a broad and flat area of the jugal bone and from the postero-dorsal area of the arch posterior to the orbit (Figs. 5G and 5H). The fibres of the anterior portion are oriented at 30–40° to the horizontal and insert along the anterior border of the ascending ramus. The posterior portions run vertically and insert on the lateral surface of the ascending ramus superior to the insertion of the deep masseter. The posterior medial surface of this muscle is in close contact to the temporalis and separates from it anteriorly.

#### Temporalis complex

**Figure 6:**
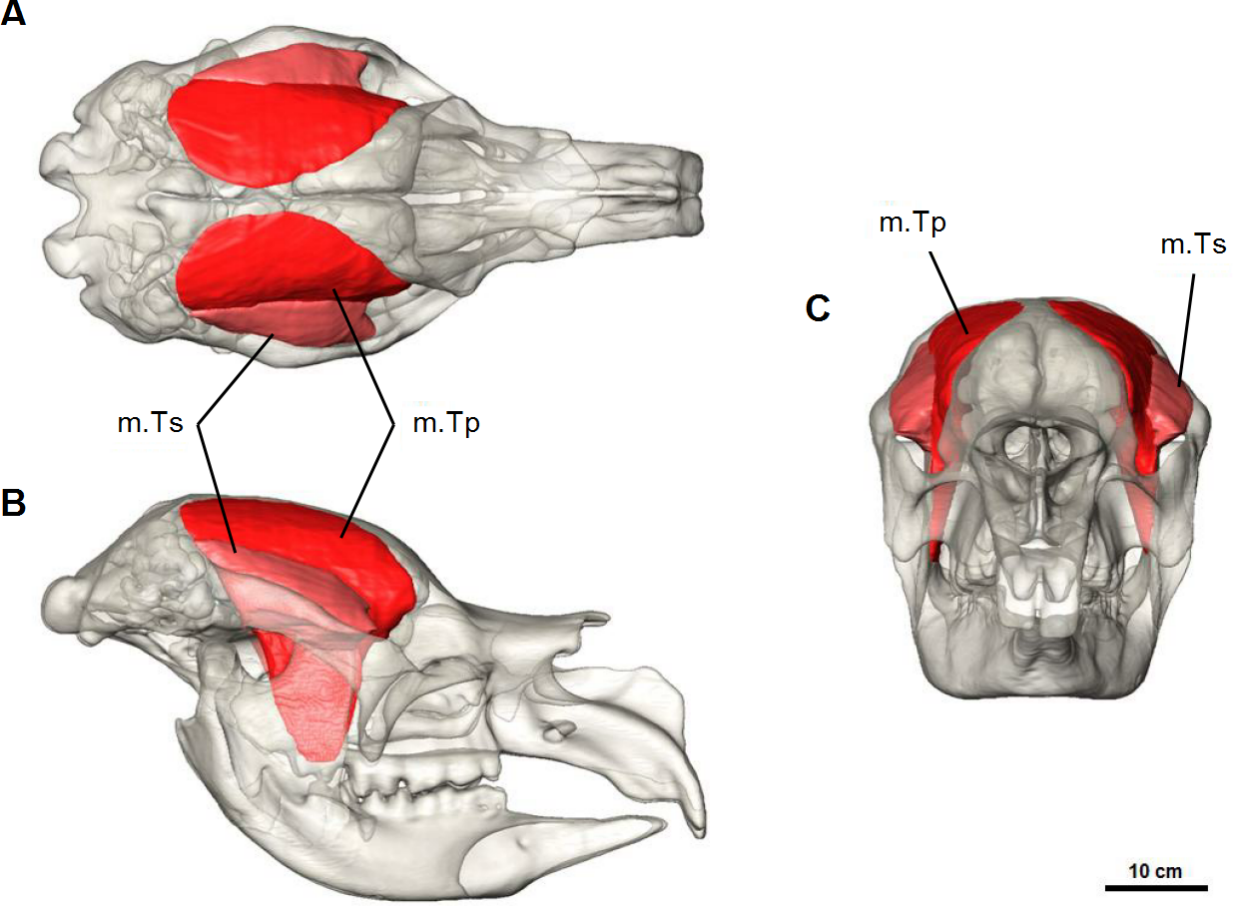
Temporalis muscle reconstruction for *Diprotodon*. (A) Dorsal, (B) lateral and (C) anterior views. Abbreviations: m.Ts, superficial temporalis; m.Tp, deep temporalis.

The temporalis muscle completely fills the temporal area from the zygomatic arch to the dorsal surface of the frontal and parietal bones (Fig. 6). This is indicated by moderately developed frontal crests that extend beyond the fronto-parietal suture and converge at the dorsal midline. From previous descriptions of the muscle in modern marsupials (Crompton et al., 2008; Davison & Young, 1990; Tomo et al., 2007; Warburton, 2009), and from additional dissections, the temporalis was reconstructed as two parts: the superficial lateral part (m.Ts) and the deep medial part (m.Tp), following (Warburton, 2009). The smaller lateral portion of the temporalis originates from the postero-medial portion of the zygomatic arch and squamosal bone, and inserts to the superior-lateral border of the coronoid process of the mandible. The deep medial temporalis was the largest individual masticatory muscle portion reconstructed in *D. optatum* (Table 1) and has an extensive origin on the frontal, parietal, and squamosal bones. It is bordered posteriorly by the occipital crest, dorsally by the midsagital plane and runs diagonally forward and laterally, following the faint temporal line to the postorbital process of the frontal bone. It inserts on the superior and anterior edges of the coronoid process of the mandible, and to the medial surface of the ascending ramus.

**Table 1:** Estimated volume, and percentage of volume, for each reconstructed muscle in the Bacchus Marsh Diprotodon.

Muscle	Volume (mm^3^)	Percentage of total volume
Deep Temporalis (m.Tp)	1,551,705	35
Superficial Temporalis (m.Ts)	366,344	8
***Total Temporalis***	***1,918,049***	***43***
Superficial Medial Pterygoid (m.PTms)	99,546	2
Deep Medial Pterygoid (m.PTmp)	409,059	9
Lateral Pterygoid (m.PTl)	73,071	2
***Total Pterygoid***	***581,677***	***13***
Zygomaticomandibular (m.ZM)	705,058	16
Deep Masseter (m.Mp)	512,864	11
Superficial Masseter (m.Ms)	757,080	17
***Total Masseter***	***1,975,002***	***44***
***Total***	***4,474,728***	***100***

The primary function of the temporalis muscles is to raise and retract the mandible. The anterior fibres act to elevate the mandible whilst the posterior fibres provide a backward pull during jaw closure.

#### Pterygoid complex

**Figure 7:**
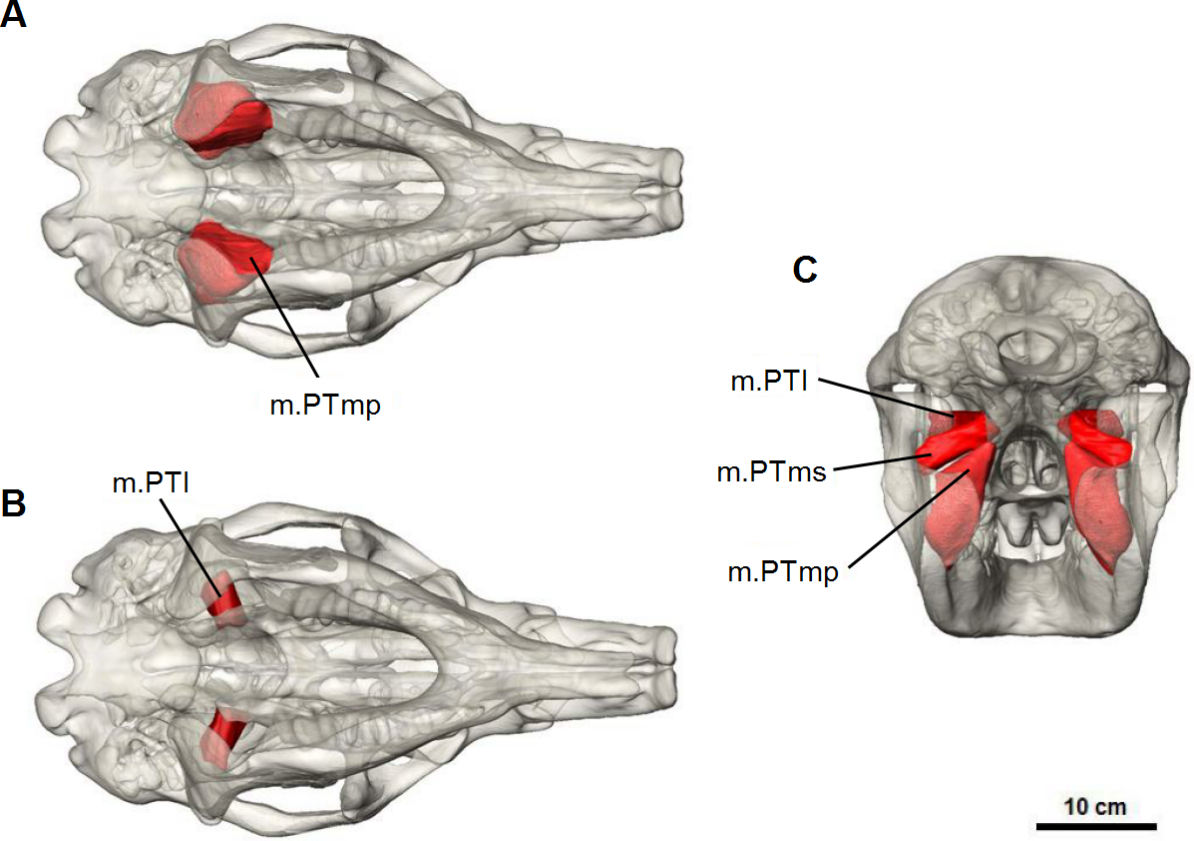
Pterygoid muscle reconstruction for *Diprotodon*. (A, B) ventral views, (C) posterior view. Abbreviations: m.PTl, lateral pterygoid; m.PTms, superficial medial pterygoid; m.PTmp, deep medial pterygoid.

*M. pterygoideus medialis* (m.PTm) is divided into two parts: a deep part (m.PTmp) and a superficial part (m.PTms) (Fig. 7). The deep part originates from the ventral edge of the pterygoid bone and inserts on the medial edge and anterior surface of the inflected angle of the mandible. The superficial part has an area of origin in the pterygoid fossa, a concave pocket on the lateral surface of the alisphenoid bone formed by the descending process and lateral wing. It inserts on the medial surface of the ascending ramus, ventral to the articular process. The medial pterygoid works in combination with the masseter and anterior fibres of temporalis to elevate the mandible.

*M. pterygoideus lateralis* (m.PTl) is a short cylindrical muscle which originates from the lateral surface of the alisphenoid, dorsal to the medial pterygoid. The origin site is smooth and not marked by clear muscle scars or ridges. This muscle inserts on the medial aspect of the condyle and articular disc. Due to its postero-lateral direction of action, the lateral pterygoid would provide transverse movements of the lower jaw.

## Discussion

Murray (1992) concluded that many marsupial megafauna probably had a “Generalized” muscle structure, in being intermediate between the “Specialized” forms outlined by Turnbull (1970). Turnbull’s Generalized Group is characterised by a dominant temporalis, which on average amounts to over 50% of the total jaw adductor musculature, and a masseter ranging between 22 and 35% of the total mass. Mammals placed in this category are relatively unspecialised and represent the basic, primitive condition of mammals like *Didelphis* (Turnbull, 1970). The three specialised groups include the “carnivore-shear” type (Group I) characterised by a simple hinge movement of the jaw and a highly developed temporalis muscle; the “ungulate-grinding” type (Group II) typified by the ungulates that grind food with a medio-lateral direction and have a relatively small temporalis, large masseter and pterygoids; and, the “rodent-gnawing” or “anterior shift” type (Group III) that have a dominant masseter and an anterior movement of the lower jaw to occlude the incisors. Murray (1992) placed Diprotodontid and Palorchestid marsupials in the Generalized Group based on large temporalis fossae and surface attachment areas for the temporalis, measured from 2D views, which amounted to more than 50% of the total adductor complex. However, basing the volume or mass of a muscle group on a 2D lateral view does not take into account the large inflected angle of the mandible, or the flared masseteric ridge for attachment of the pterygoid and masseter muscle groups. The 3D muscle reconstruction performed for this study represents the full volume of the muscle groups and offers a more precise estimate of the extent of each muscle, permitting comparisons with muscles weights from Turnbull (1970). The relative proportions for each muscle group in *Diprotodon* differ from muscle proportions considered as the standard Generalized Group in Turnbull. In *Diprotodon* the masseter and temporalis are essentially equal in size, varying only by 1%. Therefore, the temporalis muscle cannot be considered as the dominant jaw muscle. The masseter in *Diprotodon* is also 10% larger than the maximum limit for the masseter in the Generalized Group. This configuration fits within the range for the Specialized Group II “ungulate-grinding” system, which is dominated by the masseter with an average of 48% and a range between 30 and 60%. The range for the temporalis and pterygoid muscle groups in the Specialized Group II system is also very broad (13–44% and 23–40% respectively), with the contribution alternating significantly between species, so that neither one is consistently ranked second. The temporalis muscle in *Diprotodon* is in the upper limit for the Specialized Group II system. The pterygoid muscles, however, only contribute 13% to the total jaw muscle volume and are considered small for the Specialized Group II system, but large for the Specialized Group I system in carnivores (average 8%) (Turnbull, 1970). The relatively small lateral pterygoid in *Diprotodon* and the relatively large temporalis, when compared with the Specialized Group II system, could be due to a limited lateral movement of the jaw. The transition to a herbivorous diet, and the crushing movement that is essential for processing vegetation, could explain the divergence from a Generalized Group muscle structure, found in more basal marsupials that were not processing large portions of vegetation.

The masseter muscle group in *Diprotodon* reflects similarities and differences to the masseter of macropods and wombats. *Diprotodon*, like macropods, had a well developed descending process of the zygomatic arch, which is absent in the koala and wombat. The superficial layer of the masseter originates from this process and provides the anterior movement observed in macropods. Thus, it is reasonable to assume that the jaw of *Diprotodon* could also function in the same way (Sanson, 1980; Sanson, 1989; Tomo et al., 2007). Wombats lack the descending process of the zygomatic arch, but the superficial masseter is attached to the maxillo-jugal region, located anterior to the orbit and the premolar. The antero-posterior vector of muscle force provided by the superficial masseter may have functioned to provide fine control at the incisors, rather than crushing force along the molars, differing from the other masseter muscles. This fine control could be used to selectively pick leaves or grass. Both the upper and lower incisors of *Diprotodon* are open-rooted, like wombats, allowing continuous growth throughout the animal’s life. Therefore, the incisors must undergo considerable use when processing vegetation. It may be possible that *Diprotodon* occasionally stripped bark from trees, as has been observed in wombats (Triggs, 2009).

It also appears that the medial pterygoid in *Diprotodon* had undergone hypertrophy and specialization, while the lateral pterygoid remained small. Unlike koalas (Davison & Young, 1990), the large inflected angle of the mandible present in *Diprotodon*, kangaroos and wombats, provides an area of insertion for the medial pterygoid. The medial pterygoid accounts for 11% of the total masticatory muscle mass in *Diprotodon* and likely worked in combination with the masseter and anterior fibres of temporalis to elevate the mandible, producing a vertical movement. While the medial pterygoid would still provide some transverse force, the reduction of the lateral pterygoid means there was only a small force for transverse movements of the jaw. It is, therefore, likely that the main jaw movement in *Diprotodon* was vertical, providing a crushing action at the molars.

The masticatory motor program, or sequence of activity of the jaw muscles during chewing, in *Diprotodon* is impossible to know. However, comparisons with other marsupials could shed more light on the jaw movements of *Diprotodon*. In all extant diprotodonts, jaw movements consist of a vertical Phase I and transverse Phase II stroke (Crompton, 2011). However, the sequence of activation of the muscles changes considerably between species. Wombats are unique in that the power stroke is restricted to a single transverse phase where only the muscles on the working side are active (Crompton et al., 2008). In contrast, macropods and koalas exhibit a vertical phase followed by a transverse phase of varying degrees (Crompton, 2011; Crompton, Owerkowicz & Skinner, 2010). Transverse movements of the jaw came late in the evolution of macropods which occurred with a shift to a grazing diet. Within Macropodoidea, potoroos retain the primitive linear vertical closing stroke and limited transverse movement of the jaw, with near synchronous activation of the balancing and working side jaw muscles (Crompton, 2011). Based on the similarity of molar structure, skull and jaw morphology and muscle proportions, it is probable that *Diprotodon* had a similar mode of feeding to macropods, with a dominant Phase I consisting of a powerful vertical stroke followed by a moderate transverse movement powered by the working-side superficial masseter and medial pterygoid, rather than the more dominant Phase II transverse movement observed in wombats.

Interpretations of the feeding ecology and diet of *Diprotodon* have mostly been based on an isotope analyses of the teeth to determine C3/C4 plant preference (Gröcke, 1997). However, no in-depth analysis has been done on macro- or micro-wear, or a morphological assessment of the teeth to assess diet or the mode of feeding. The family to which *Diprotodon* belongs, Diprotodontidae, has no living representatives. The closest living relatives to *Diprotodon* are the wombats and koalas from the families Vombatidae and Phascolarctidae respectively. However, the tooth morphology of *Diprotodon* is more similar to that of macropods, which have relatively simple bilophodont molars (Sanson, 1989). The molars of macropods reflect a range of diets from mixed invertebrate, seed and low plant fibre diet, to browser grade (low fibre browse), to grazer grade (grass diet) and intermediates between grazers and browsers (Sanson, 1989). Grazing macropods, such as those from the genus *Macropus*, have modified their molars and incisors to crop and break down tough grasses which compose 90% of their diet (Sanson, 1980; Sanson, 1989). They have evolved tall transverse lophs adapted to shear food as the jaw moves vertically, and to crush food between the lophs and the well-developed links as the jaw moved transversely (Crompton, 2011; Sanson, 1980). All grazing kangaroos also exhibit some degree of molar progression, a reduced premolar and a dorsally convex curve of the lower tooth row so that the whole tooth row is never in contact at one time. Worn teeth are removed from the occlusal plane and are gradually replaced by unworn teeth that move anteriorly in the mandible and dorsally into the occlusal plane. As a tooth becomes so worn that it is useless, it moves anteriorly and ventrally out of the occlusal plane. These features correlate with a predominant diet of tough grass (Bensley, 1903; Ride, 1959; Sanson, 1980; Sanson, 1989).

In contrast, browsing wallabies, such as the Swamp Wallaby (*Wallabia bicolor*), have simpler molars without strong links between the lophs, a well-developed premolar, a flat tooth row and no molar progression (Sanson, 1980; Sanson, 1989). *W. bicolor* has a crushing action at the molars for softer material, and there is no need to replace worn teeth because worn molars retain some useful function. The lower tooth row remains flat so that all teeth occlude in the adults. The molars of *Diprotodon* are simple bilophodont molars without strong links and the occlusal surface of the upper molars is slightly convex downwards, but not to the high degree seen in *Macropus*. With wear, the enamel gains thickness giving more resistance or grinding-power as the tooth wears down, and then thins again with extreme wear toward the base of the lobe. There is no evidence of molar progression but heavier wear does occur on the anterior molars, so that when the posterior molars erupt, the first and second molars are already quite worn (Owen, 1870). Thus, it’s likely that *Diprotodon* had a mixed diet similar to that of browsing wallabies rather than one dominated by grass like the diet of grazing kangaroos. This supports the hypothesis that *Diprotodon* was an opportunistic intermediate browser/grazer, capable of changing its diet with changing environmental conditions.

The skull of *Diprotodon* had large sinuses, a small brain and a muscle structure that was moving away from that of marsupials within the Generalized Group and toward a more specialised structure for processing vegetation. The reconstructed masseter and temporalis muscles are approximately equal in size, both functioning together to elevate the lower jaw. Lateral movement of the lower jaw was limited by the small size of the lateral pterygoid, with the medial pterygoid becoming larger to support the function of the temporalis and masseter muscles. The dominant vertical movement of the jaw would have provided a crushing action at the molars, also facilitated by the prominent lophs and absence of links in M1–M4. The antero-lateral origin of the superficial masseter on the descending process of the zygomatic arch may indicate that antero-posterior movement in the lower jaw provided fine control at the incisors. The large sinuses in *Diprotodon* may have expanded to compensate for the small size of the braincase, and provide attachment for the temporalis muscles, which is relatively large when compared to placental herbivores that belong in the Specialized Group II, characteristic of the “ungulate-grinding” system.
